# Microdosing as a Potential Tool to Enhance Clinical Development of Novel Antibiotics: A Tissue and Plasma PK Feasibility Study with Ciprofloxacin

**DOI:** 10.1007/s40262-021-01091-1

**Published:** 2022-01-07

**Authors:** Zoe Oesterreicher, Sabine Eberl, Beatrix Wulkersdorfer, Peter Matzneller, Claudia Eder, Esther van Duijn, Wouter H. J. Vaes, Birgit Reiter, Thomas Stimpfl, Walter Jäger, Alina Nussbaumer-Proell, Daniela Marhofer, Peter Marhofer, Oliver Langer, Markus Zeitlinger

**Affiliations:** 1 Department of Clinical Pharmacology, Medical University of Vienna, Währinger Gürtel 18-20, 1090 Vienna, Austria; 2 Internal Medicine 2, Gastroenterology and Hepatology and Rheumatology, University Hospital of St. Pölten, St. Pölten, Austria; 3 TNO, Zeist, The Netherlands; 4 Department of Laboratory Medicine, Medical University of Vienna, Vienna, Austria; 5 Department of Pharmaceutical Sciences, University of Vienna, Vienna, Austria; 6 Department of Anaesthesia, General Intensive Care and Pain Therapy, Medical University of Vienna, Vienna, Austria; 7 Orthopaedic Hospital Speising, Vienna, Austria

## Abstract

**Background and Objective:**

In microdose studies, drug pharmacokinetics is measured in humans after administration of subtherapeutic doses. While previous microdose studies focused primarily on plasma pharmacokinetics, we set out to evaluate the feasibility of microdosing for a pharmacokinetic assessment in subcutaneous tissue and epithelial lining fluid.

**Methods:**

Healthy subjects received a single intravenous bolus injection of a microdose of [^14^C]ciprofloxacin (1.1 µg, 7 kBq) with (cohort A, *n* = 9) or without (cohort B, *n* = 9) a prior intravenous infusion of a therapeutic dose of unlabeled ciprofloxacin (400 mg). Microdialysis and bronchoalveolar lavage were applied for determination of subcutaneous and intrapulmonary drug concentrations. Microdose [^14^C]ciprofloxacin was quantified by accelerator mass spectrometry and therapeutic-dose ciprofloxacin by liquid chromatography–tandem mass spectrometry.

**Results:**

The pharmacokinetics of therapeutic-dose ciprofloxacin (cohort A) in plasma, subcutaneous tissue, and epithelial lining fluid was in accordance with previous data. In plasma and subcutaneous tissue, the dose-adjusted area under the concentration–time curve of microdose ciprofloxacin was similar in cohorts A and B and within an 0.8-fold to 1.1-fold range of the area under the concentration–time curve of therapeutic-dose ciprofloxacin. Penetration of microdose ciprofloxacin into subcutaneous tissue was similar in cohorts A and B and comparable to that of therapeutic-dose ciprofloxacin with subcutaneous tissue-to-plasma area under the concentration–time curve ratios of 0.44, 0.44, and 0.38, respectively. Penetration of microdose ciprofloxacin into epithelial lining fluid was highly variable and failed to predict the epithelial lining fluid penetration of therapeutic-dose ciprofloxacin.

**Conclusions:**

Our study confirms the feasibility of microdosing for pharmacokinetic measurements in plasma and subcutaneous tissue. Microdosing combined with microdialysis is a potentially useful tool in clinical antimicrobial drug development, but its applicability for the assessment of pulmonary pharmacokinetics with bronchoalveolar lavage requires further studies.

**Clinical Trial Registration:**

ClinicalTrials.gov NCT03177720 (registered 6 June, 2017).

**Supplementary Information:**

The online version contains supplementary material available at 10.1007/s40262-021-01091-1.

## Key Points

**Table:** 

In several drug development scenarios, microdose studies have provided valuable information to improve preclinical drug candidate selection.
While previous microdose studies have so far mainly focused on assessing drug pharmacokinetics in plasma, we assessed whether microdosing with the ^14^C-labeled antibiotic ciprofloxacin as a model drug can be used in combination with microdialysis and bronchoalveolar lavage for determination of tissue pharmacokinetics.
Our study showed that the combination of microdosing with ^14^C-labeled drug and microdialysis was able to predict the tissue pharmacokinetics of a therapeutic dose of ciprofloxacin.
Our study extends the scope of microdose studies to the assessment of drug tissue pharmacokinetics, thereby potentially providing an attractive alternative approach for drug development, which saves time and costs.

## Introduction

The urgent need for new antimicrobial agents because of antimicrobial resistance is omnipresent; however, antibiotic development is hindered by high costs and time. Preclinical studies take about 5.5 years and cost millions of dollars, and only 9% of substances in preclinical studies succeed [1]. Novel faster means of approval are sought by pharmaceutical companies and are supported by regulatory bodies. The concept of microdosing, also termed as phase 0 studies, emerged in the early 1990s and comprises the administration of a single subtherapeutic dose (< 1/100th of the pharmacologically active dose of the test substance calculated from animal studies) of a drug candidate to humans in order to describe the drug’s pharmacokinetic (PK) profile [2, 3]. Because of the low doses generally used (often microdoses), phase 0 studies require more sensitive analytical tools than conventional PK studies, such as accelerator mass spectrometry (AMS), positron emission tomography (PET), or liquid chromatography/tandem mass spectrometry (LC–MS/MS) [4]. Because of the small dose administered (usually ≤ 100 µg for small molecules and ≤ 30 nmol for protein products), study participants are exposed to a minimal risk [5, 6]. As less preclinical testing is required, microdose studies can be performed earlier during drug development, thereby potentially saving time and costs [2, 7].

Actual efficacy of an antimicrobial substance is limited by its penetration to the site of infection. A constantly increasing number of studies evaluates tissue penetration using microdialysis or bronchoalveolar lavage (BAL). Microdialysis is a minimally invasive method that allows the quantification of an unbound drug concentration in interstitial fluid. Microdialysis for the determination of target-site pharmacokinetics during early drug development on the one hand, or for the optimization of current dosing regimens on the other hand, has become an important tool in PK studies [8, 9]. While microdialysis allows the determination of, for example, cerebral [10, 11] or peripheral soft-tissue concentrations [12–18], its application for lung PK determination would require a thoracotomy [19]. Performing BAL enables the quantification of drug pharmacokinetics in epithelial lining fluid (ELF) [12, 20–22].

Despite the importance of tissue pharmacokinetics, microdose PK studies using AMS are mainly limited to assessing drug concentrations in plasma. However, PET imaging provides information on the tissue distribution of radiolabeled drugs administered at microdoses, but measures total (i.e., unbound and bound) drug concentrations and therefore fails to describe the microbiologically active fraction of an antibiotic [23–26]. Positron emission tomography requires complex research infrastructure as the study drug has to be radiolabeled on-site with short-lived positron-emitting radionuclides, such as carbon-11 (half-life: 20.4 minutes) or fluorine-18 (half-life: 109.8 minutes) [25].

The present study for the first time set out to evaluate the feasibility of microdosing in combination with microdialysis and BAL for predicting target-site pharmacokinetics of therapeutic doses. We selected the fluoroquinolone antibiotic ciprofloxacin as a model drug because of our previous experience in assessing the tissue distribution of this drug with microdialysis and PET in humans [23, 27–29]. A microdose of carbon-14- (^14^C, half-life: 5730 years) labeled ciprofloxacin ([^14^C]ciprofloxacin) was administered to healthy volunteers as a single intravenous (i.v.) dose with or without prior administration of a therapeutic dose of unlabeled ciprofloxacin, and plasma, subcutaneous, and ELF concentrations were quantified by AMS and LC–MS/MS. The chosen study design additionally enabled us to assess whether administration of a therapeutic dose of ciprofloxacin exerted an influence on the pharmacokinetics of the ciprofloxacin microdose.

## Methods

This prospective single-center study was performed at the Department of Clinical Pharmacology, Medical University of Vienna, Austria and conducted in accordance with the Declaration of Helsinki and the current revisions of the Good Clinical Practice Guidelines of the European Commission and the Good Scientific Practice Guidelines of the Medical University of Vienna. The protocol was approved by the Ethics Committee of the Medical University of Vienna (EK Number 1542/2015, EudraCT Number 2015-002611-15) and the national competent authority (Bundesamt für Sicherheit im Gesundheitswesen). Informed consent was sought from all healthy volunteers. Eighteen healthy male volunteers were included in this trial. Inclusion criteria were age between 18 and 55 years, non-smoker, normal vital signs, and a good state of health. None of the volunteers had received a concomitant medication within 1 week or a blood donation within 4 weeks before the study.

### Study Drugs

Ciprofloxacin was obtained via the hospital pharmacy from Bayer Austria (400 mg/200 mL infusion solution). [^14^C]Ciprofloxacin (labeled in the 2-position of the quinoline ring, molar activity: 2.2 GBq/mmol, radiochemical purity: 98.2%) was obtained from Hartmann Analytic GmbH (Braunschweig, Germany). For the i.v. injection, [^14^C]ciprofloxacin (7 kBq, 1.1 µg) was dissolved in 10 mL of physiological saline solution and filtered through a sterile Millex-GS filter (0.22 µm; Millipore Corporation, Bedford, MA, USA).

No formal dosimetry calculation was performed for [^14^C]ciprofloxacin. Because of the very high sensitivity of AMS, the administration of only very low ^14^C amounts is required (7 kBq in the current study). In addition, as the energy of the β^−^ particles emitted in the decay of ^14^C is relatively low (0.156476 MeV), AMS microdosing studies usually fall within risk category I (trivial risk, total effective dose < 0.1 mSv) according to the International Commission on Radiological Protection Publication 62 “Radiological Protection in Biomedical Research” [30]. However, an intravenously injected ^18^F-labeled radiotracer for PET (400 MBq) typically gives a total effective dose in the range of 5–10 mSv, corresponding to risk category IIb (minor to intermediate risk) in the International Commission on Radiological Protection publication 62 [30].

### Microdialysis

Ciprofloxacin concentrations were determined in subcutaneous tissue using microdialysis. This method allows quantification of the unbound fraction of a substance in interstitial space fluid by insertion of a microdialysis catheter into the tissue of interest. The microdialysis catheter is equipped with a semipermeable membrane at the tip and is constantly perfused at a predefined flow rate with physiologic solution [8, 9]. CMA 66 catheters (M Dialysis AB, Solna, Sweden) having a membrane with a molecular weight cut-off of 20 kDa were used in this study and perfused at a flow rate of 1.0 µL/min. The ciprofloxacin concentration that penetrated the semipermeable membrane via passive diffusion from the tissue into the dialysate was determined (*C*_dialysate_). Because of a lack of equilibrium between extracellular tissue fluid and the perfusion medium, a probe calibration was performed after the PK sampling period using the retrodialysis method. This method assumes that the exchange process across the semipermeable membrane of the microdialysis probe is equal in both directions. After at least 30 minutes of equilibration, the catheter was perfused with a known concentration of either unlabeled ciprofloxacin (5 µg/mL) or [^14^C]ciprofloxacin (10.5 pg/mL) for 1 hour at a flow rate of 1.0 µL/min and the concentration of ciprofloxacin in the dialysate was determined. In vivo recovery (%) was calculated as 100 − (100 . ciprofloxacin concentration_dialysate_ /ciprofloxacin concentration_perfusate_). The concentration in the interstitial tissue was then determined as *C*_dialysate_/in vivo recovery.

### Bronchoalveolar Lavage (BAL)

Bronchoalveolar lavage relies on the concept that by rinsing the bronchoalveolar tree with saline, ELF can be collected [12]. Epithelial lining fluid samples were taken using BAL at 2 h, 4 h, or 8 h after [^14^C]ciprofloxacin administration. Because of the invasiveness of the procedure, each volunteer underwent only one BAL procedure, and timepoints were randomly assigned using envelopes to the subjects in order to obtain three samples per time point. Sedation was performed using propofol and remifentanil while vital parameters were monitored constantly. The airway was secured with a laryngeal mask and anesthesia was maintained with 1 MAC sevoflurane. After administering xylocaine^®^ 2% gel at the *rima glottides*, BAL was performed by instilling three 20-mL aliquots of saline solution.

### Urea Dilution Method

As each BAL sample consisted of ELF in saline, the dilution of ELF was determined to convert ciprofloxacin concentrations in BAL fluid to ciprofloxacin concentrations in ELF. The urea dilution method was used to assess ELF dilution [31]. Urea freely diffuses through several body compartments, including ELF, as supported by experiments in the isolated perfused dog lung [32]. If the concentrations of urea in plasma (*C*_urea,plasma_) and BAL fluid (*C*_urea,BAL_) are known, concentrations of ciprofloxacin in BAL samples (*C*_ciprofloxacin,BAL_) can be corrected for dilution of ELF by saline to obtain ciprofloxacin concentrations in ELF (*C*_ciprofloxacin,ELF_) according to the following equation:

*C*_ciprofloxacin,ELF_ = *C*_ciprofloxacin,BAL_/(*C*_urea,BAL_/*C*_urea,plasma_)

To assess plasma concentrations of urea, one plasma sample was drawn near to the timepoint of BAL. The concentration of urea in both plasma and BAL fluid was determined using a specific urea assay kit (Abnova KA1652) according to the manufacturer’s protocol. Urea reactions were performed in 96-well plates and concentrations were assessed using an EnSpire^®^ 2300 Multimode Plate Reader (PerkinElmer, Waltham, MA, USA) at an absorbance of 520 nm. All standards and samples were run in duplicate and the mean values were used for analysis. The limit of quantification for plasma and BAL was 0.8 µg/mL.

### Study Days and Procedures

Healthy subjects underwent a screening visit, one study day, and a final visit. The 18 healthy subjects were randomly assigned using envelopes to one of two cohorts, each consisting of nine subjects. Both cohorts received a microdose of [^14^C]ciprofloxacin (7 kBq, 1.1 µg, dissolved in 10 mL of physiological saline solution) as an i.v. bolus over approximately 1 min. In cohort A, the [^14^C]ciprofloxacin microdose administration was preceded by a single i.v. infusion of a therapeutic dose of unlabeled ciprofloxacin (400 mg) over 60 min, while cohort B only received the [^14^C]ciprofloxacin microdose. On the study day, one microdialysis catheter was inserted into the subcutaneous tissue of the upper thigh, followed by the study drug administration. The i.v. bolus of the microdose of [^14^C]ciprofloxacin was administered immediately after the end of the infusion of the therapeutic dose of unlabeled ciprofloxacin. Blood sampling was subsequently performed at 0.5, 1, 1.5, 2, 2.5, 3, 4, 5, 6, 7, 8, and 10 hours after administration using lithium heparin tubes (Vacuette^®^ FX; Greiner Bio-One, Kremsmünster, Austria). Samples were centrifuged (2000*g*, 4 °C, 10 min) within 1 hour after collection, snap frozen, and stored at −80 °C for determination of ciprofloxacin concentrations using LC–MS/MS. Microdialysates were sampled three times over 1 hour, respectively (from 1.5 to 2.5 h, from 3.5 to 4.5 h, and from 7.5 to 8.5 h after [^14^C]ciprofloxacin administration). Retrodialysis was performed after at least 30 min of equilibration and retrodialysis samples were collected from 0 to 0.5 h and from 0.5 to 1 h. Microdialysis samples were snap frozen and stored at – 80 °C for determination of ciprofloxacin concentrations using LC-MS/MS and AMS. Bronchoalveolar lavage was performed in each subject at one of three timepoints after [^14^C]ciprofloxacin administration (2, 4, or 8 h) as described above. The aliquots of BAL fluid were pooled, centrifuged (2000*g*, 4 °C, 10 min) and the supernatant was snap frozen and stored at −80 °C for determination of ciprofloxacin concentrations using LC–MS/MS and AMS. At the time of BAL, one blood sample was collected, which was used for determination of urea concentrations (see above) and for determination of ciprofloxacin concentrations in plasma using AMS.

### Chemical Analysis

#### LC–MS/MS Analysis

Liquid chromatography tandem mass spectrometry analysis of ciprofloxacin in plasma, microdialysis, and BAL fluid samples was performed at the Department of Laboratory Medicine (Medical University of Vienna, Vienna, Austria) as described in the Electronic Supplementary Material.

#### AMS Analysis

Accelerator mass spectrometry analysis was conducted at TNO (Zeist, The Netherlands). As ciprofloxacin is hardly metabolized (i.e., at 2 h after an i.v. bolus injection of [^18^F]ciprofloxacin no radiolabeled metabolites could be detected in plasma [23]; after an i.v. infusion of [^18^F]ciprofloxacin, > 85% of total radioactivity excreted into urine over 5 h was in the form of unchanged [^18^F]ciprofloxacin [27]), total ^14^C content was quantified in plasma, microdialysis, and BAL fluid samples without prior chromatographic separation. Total radioactivity analysis by AMS in various matrices has been qualified [33]. The qualification is independent of the ^14^C-labeled compound and can therefore be used in any radiolabeled study. Accelerator mass spectrometry is a highly sensitive technology that requires particularly low sample volumes, providing new possibilities in bioanalysis. The only critical parameters in quantifying total radioactivity levels are the amounts of ^14^C and ^12^C, which together determine the ^14^C/^12^C ratio measured. For matrices that contain too limited amounts of ^12^C, the sample is supplemented with ^12^C to a similar ^12^C level as naturally present in plasma samples. The supplementation is done by the addition of acetaminophen, which is required for a stable ^14^C/^12^C ratio analysis. For each microdialysis and BAL fluid sample, a volume of 5 µL was transferred to a tinfoil cup to which subsequently a solution of acetaminophen in methanol (16 µL, 19.7 mg/mL) was added. For each plasma sample, a volume of 5 µL was transferred to a tinfoil cup. The tinfoil cups were dried under a stream of nitrogen and subsequently combusted on an elemental analyzer (Vario Micro, Elementar, Germany). Generated CO_2_ was transferred to a home-built gas interface, composed of a zeolite trap and syringe [33]. CO_2_ was adsorbed to the trap on the interface. After heating of the trap, the CO_2_ was transferred to a vacuum syringe using helium. A final CO_2_/helium mixture of 6% was directed to the AMS ion source, at a pressure of 1 bar and a flow rate of 60 µL/min. A 1 MV Tandetron AMS (High Voltage Engineering Europe B.V., Amersfoort, The Netherlands) was used.

### PK Calculations

Total ^14^C concentrations measured with AMS were assumed to equal the concentrations of [^14^C]ciprofloxacin. [^14^C]Ciprofloxacin concentrations in plasma and ELF and therapeutic-dose ciprofloxacin concentrations in ELF were only measured at one timepoint per subject (i.e., at 2, 4, or 8 h after administration) and concentrations from three subjects per timepoint were averaged to generate concentration–time profiles. Concentrations of [^14^C]ciprofloxacin (mBq/mL) were converted into mass concentrations (pg/mL) via the molar activity of [^14^C]ciprofloxacin (2.2 GBq/mmol). To enable comparison of therapeutic-dose and microdose ciprofloxacin concentrations in plasma, subcutaneous tissue, and ELF, values were normalized to the administered dose of ciprofloxacin (in µg) and expressed as pg equivalents per mL. To assess correlations between dose-adjusted concentrations of microdose and therapeutic-dose ciprofloxacin, Pearson’s coefficient of correlation (*r*) was calculated.

Pharmacokinetic analysis was performed using the Kinetica 2000 software package, version 3.0 (InnaPhase, Philadelphia, PA, USA). The following parameters were calculated for plasma, subcutaneous tissue, and ELF: maximum concentration, time to maximum concentration, area under the concentration–time curve (AUC) from time 0 to 10 h for plasma, and from 0 to 8 h for subcutaneous tissue and ELF. In addition, for plasma, the elimination half-life, apparent total body clearance from plasma, and apparent volume of distribution at steady state were calculated. To quantify tissue penetration of ciprofloxacin, the subcutaneous tissue-to-plasma (AUC_ST_/AUC_plasma_) and ELF-to-plasma (AUC_ELF_/AUC_plasma_) AUC ratios were calculated. Pharmacokinetic parameters are given as geometric mean and 95% confidence interval or median and range.

## Results

Eighteen healthy male subjects completed the study (mean ± standard deviation age: 30 ± 8 years, mean ± standard deviation height: 183 ± 8 cm, mean ± standard deviation weight: 79 ± 9 kg). Nine subjects received an i.v. microdose of [^14^C]ciprofloxacin (1.1 µg) preceded by an i.v. therapeutic dose of unlabeled ciprofloxacin (400 mg) [cohort A], while nine subjects only received the microdose of [^14^C]ciprofloxacin (1.1 µg) [cohort B]. Administration of therapeutic-dose ciprofloxacin and [^14^C]ciprofloxacin was well tolerated without occurrence of serious adverse events. In 8 of 18 subjects, mild adverse events occurred, which were unrelated to the study drug. Because of a malfunction of the microdialysis catheter, microdialysis data of subject number 1 in cohort A were not available.

**Fig. 1:**
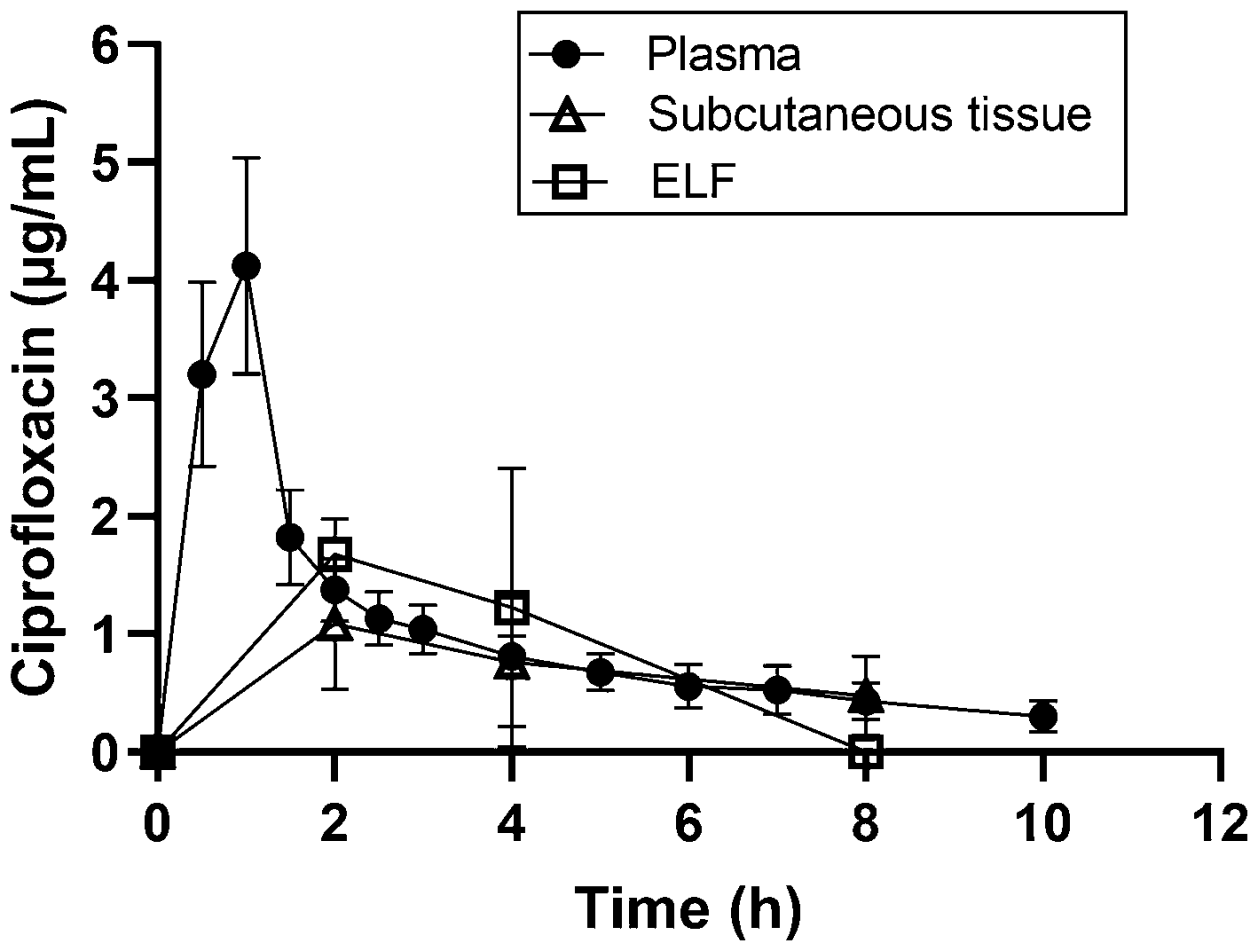
Concentration–time curves (mean ± standard deviation) of therapeutic-dose ciprofloxacin (400 mg given intravenously over 1 hour, quantified with liquid chromatography–tandem mass spectrometry, cohort A) in plasma (*n* = 9, filled circles), subcutaneous tissue (*n* = 8, open triangles), and epithelial lining fluid (ELF, open squares). Note that ciprofloxacin concentrations in ELF were determined only at one timepoint per subject and concentration–time curves represent an average of three subjects per timepoint

In Fig. 1, the concentration–time profiles of ciprofloxacin quantified with LC/MS–MS in plasma, subcutaneous tissue, and ELF after administration of the therapeutic ciprofloxacin dose (cohort A) are shown. The corresponding PK parameters of therapeutic-dose ciprofloxacin in cohort A are summarized in Table 1. Exposure to ciprofloxacin in subcutaneous tissue and ELF was 36% and 54% of that in plasma, respectively. The mean in vivo recovery for microdialysis of therapeutic-dose ciprofloxacin was 36.6 ± 12.5%.

**Table 1:** PK parameters obtained from quantification of therapeutic-dose ciprofloxacin (400 mg given intravenously over 1 hour) with liquid chromatography–tandem mass spectrometry (cohort A)

Cohort A	*t*_max_ (h)	*C*_max_ (µg/mL)	AUC ((µg/mL).h)	*t*_1/2_ (h)	CL (L/h)	*V*_ss_ (L)
Plasma	1.00 (0.50–1.00)	4.15 (3.47–4.96)	9.93 (8.58–11.50)	4.22 (3.01–5.90)	33.70 (28.38–40.02)	177.3 (139.9–224.7)
Subcutaneous tissue	2.00 (2.00–2.00)	0.95 (0.57–1.56)	3.52 (2.10–5.91)	–	–	–
ELF	2.00	1.68	5.36	–	–	–
AUC ratios	AUC_ST_/AUC_plasma_		0.36			
	AUC_ELF_/AUC_plasma_		0.54			

*t*_max_ is reported as median with range in parentheses and all other PK parameters are reported as geometric mean with 95% confidence interval in parentheses (*n* = 9 for plasma, *n* = 8 for subcutaneous tissue). For ELF, the 95% confidence interval is not given as only one timepoint per subject was measured and concentrations from three subjects per timepoint were averaged

*AUC* area under the concentration–time curve (from 0 to 10 h for plasma and from 0 to 8 h for subcutaneous tissue and ELF), *AUC*_*ELF*_*/AUC*_*plasma*_ ratio of AUC in ELF to AUC in plasma, *AUC*_*ST*_*/AUC*_*plasma*_ ratio of AUC in subcutaneous tissue to AUC in plasma, *CL* apparent total body clearance of ciprofloxacin from plasma, *C*_*max*_ maximum concentration, *ELF* epithelial lining fluid, *PK* pharmacokinetic, *t*_*1/2*_ elimination half-life, *t*_*max*_ time to maximum concentration, *V*_*ss*_ apparent volume of distribution at steady state

**Fig. 2:**
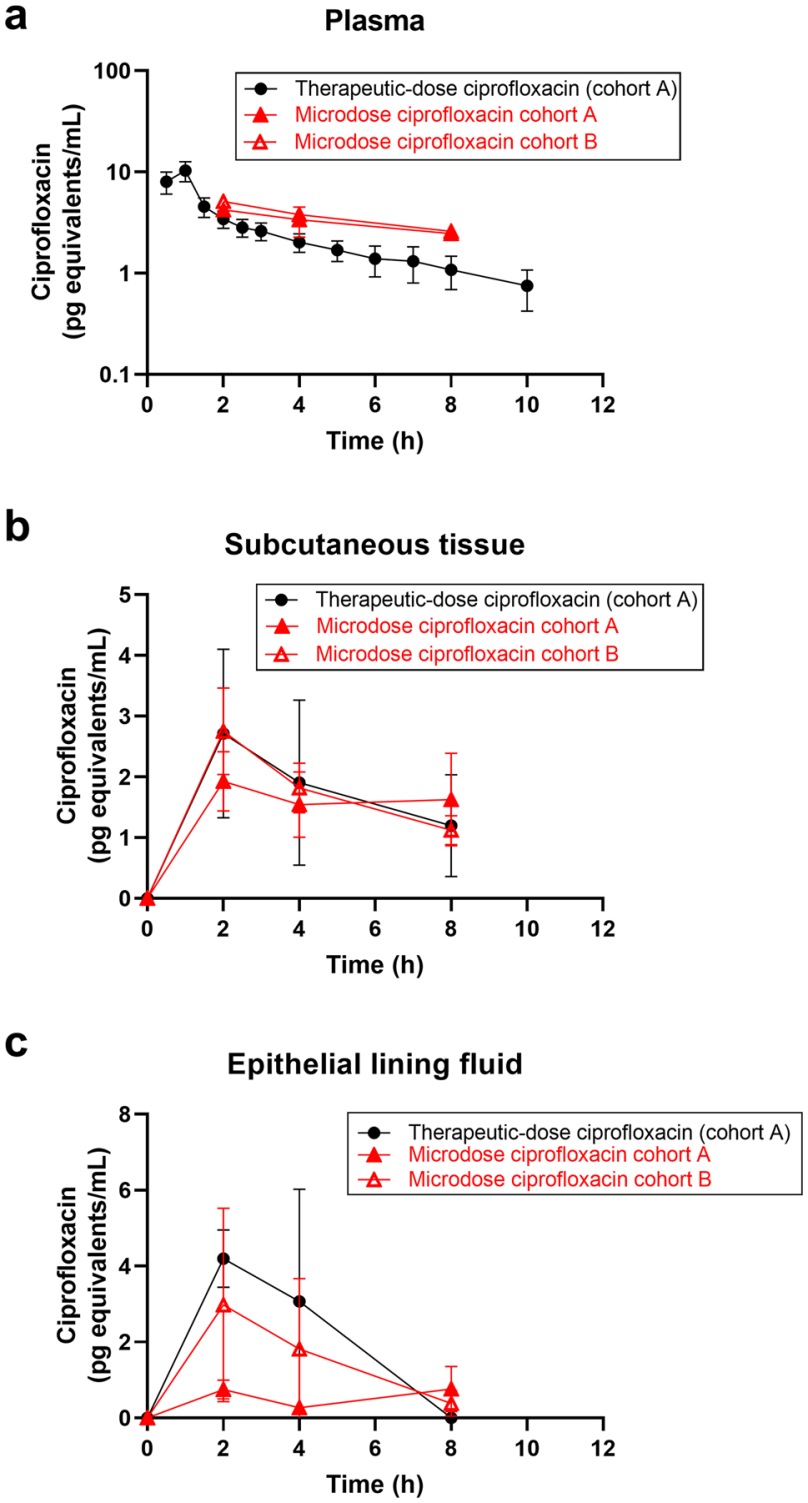
Dose-adjusted concentration–time curves (mean ± standard deviation) of therapeutic-dose ciprofloxacin (quantified with liquid chromatography–tandem mass spectrometry, cohort A) and microdose ciprofloxacin (quantified with accelerator mass spectrometry, cohort A and cohort B) in **a** plasma, **b** subcutaneous tissue, and **c** epithelial lining fluid. To enable comparison of therapeutic-dose and microdose ciprofloxacin concentrations, values were normalized to the administered dose and expressed as pg equivalents per mL. Note that both microdose and therapeutic-dose ciprofloxacin concentrations in epithelial lining fluid and microdose ciprofloxacin concentrations in plasma were determined only at one timepoint per subject and concentration–time curves represent an average of three subjects per timepoint

**Fig. 3:**
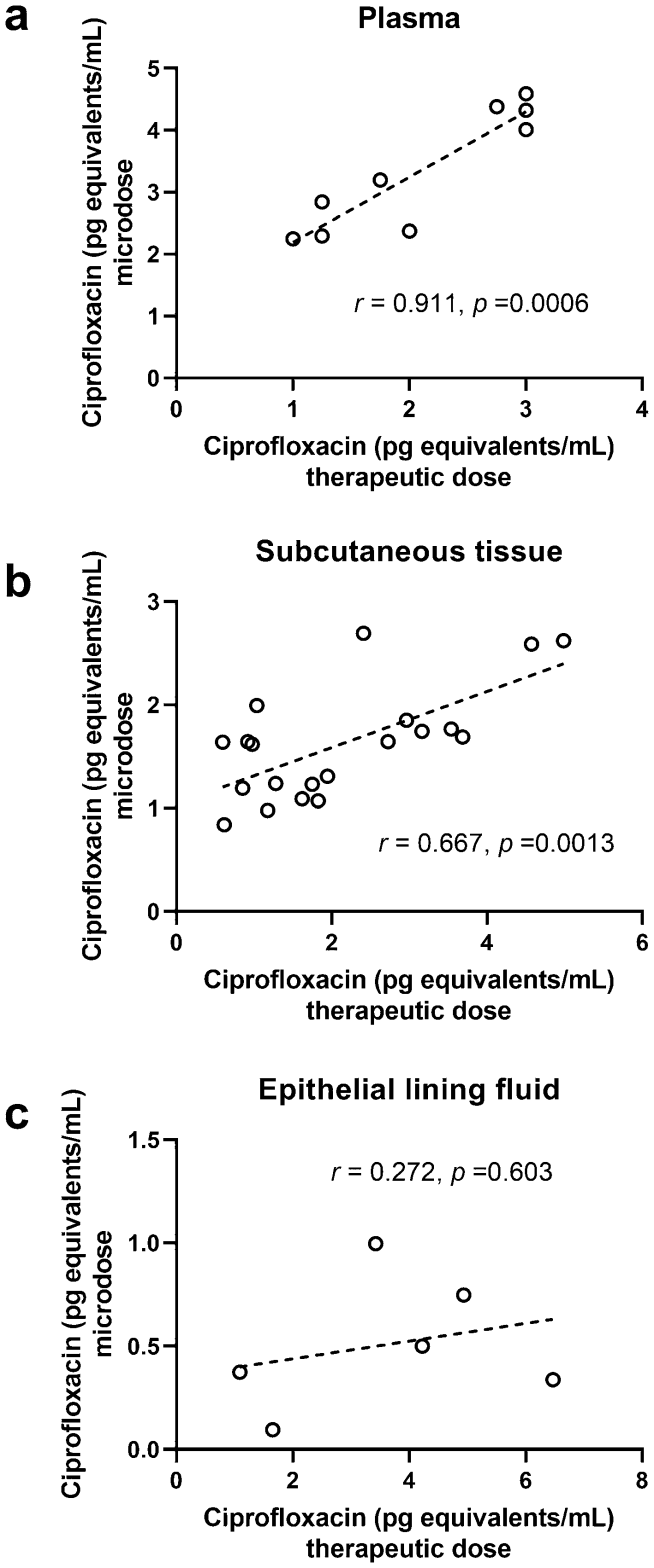
Correlations of dose-adjusted microdose ciprofloxacin concentrations determined with accelerator mass spectrometry and dose-adjusted therapeutic-dose ciprofloxacin concentrations quantified with liquid chromatography–tandem mass spectrometry in **a** plasma, **b** subcutaneous tissue, and **c** epithelial lining fluid in subjects of cohort A (*r* Pearson’s coefficient of correlation). To enable comparison of therapeutic-dose and microdose ciprofloxacin concentrations, values were normalized to the administered dose and expressed as pg equivalents per mL

To enable comparison between the pharmacokinetics of the microdose and the therapeutic dose, ciprofloxacin concentrations were normalized to the administered dose and expressed in pg equivalents/mL. In Fig. 2, the dose-adjusted concentration–time profiles of microdose ciprofloxacin in cohorts A and B quantified with AMS in plasma, subcutaneous tissue, and ELF are shown and compared with the respective dose-adjusted concentration–time profiles of therapeutic-dose ciprofloxacin measured with LC–MS/MS. The mean in vivo recovery for microdialysis of [^14^C]ciprofloxacin was 31.1 ± 12.3%, which was comparable to that of therapeutic-dose ciprofloxacin. In cohort A, concentrations of microdose ciprofloxacin in plasma and subcutaneous tissue showed a good correlation with the respective therapeutic-dose ciprofloxacin concentrations (plasma: *r* = 0.911, *p* = 0.0006; subcutaneous tissue: *r* = 0.667, *p* = 0.0013), while no correlation was observed in ELF (*r* = 0.272, *p* = 0.603) (Fig. 3).

Comparison of the dose-adjusted concentration–time profiles of microdose ciprofloxacin in plasma, subcutaneous tissue, and ELF between cohort A and cohort B revealed almost superimposable profiles in plasma (Fig. 2a) and subcutaneous tissue (Fig. 2b), while profiles diverged from each other in ELF for the two cohorts (Fig. 2c). In plasma, AMS-based concentration measurements were higher than corresponding LC–MS/MS-based concentration measurements, to a similar extent in both cohort A (1.2-fold to 2.3-fold) and cohort B (1.5-fold to 2.4-fold) (Fig. 2a). In subcutaneous tissue, microdose ciprofloxacin concentration–time profiles were almost superimposable with those of therapeutic-dose ciprofloxacin (ratio microdose/therapeutic-dose concentrations: cohort A: 0.7/1.4, cohort B: 0.9/1.0) (Fig. 2b). In ELF, microdose ciprofloxacin concentrations were markedly lower than those of therapeutic-dose ciprofloxacin in cohort A (ratio microdose/therapeutic-dose concentrations: 0.1/0.2) but in better agreement for cohort B (ratio microdose/therapeutic-dose concentrations: 0.6/0.7) (Fig. 2c). In Table 2, the dose-adjusted PK parameters of microdose ciprofloxacin are summarized for cohort A and B and compared with those of therapeutic-dose ciprofloxacin in cohort A. In plasma and subcutaneous tissue, AUC values of microdose ciprofloxacin were comparable in cohort A and B and within a 0.8-fold to 1.1-fold range of AUC values of therapeutic-dose ciprofloxacin. However, in ELF, the AUC of microdose ciprofloxacin was three-fold lower in cohort A than in cohort B and only microdose ciprofloxacin AUC in cohort B agreed reasonably well with the AUC of therapeutic-dose ciprofloxacin. The AUC_ST_/AUC_plasma_ ratios were very similar for microdose ciprofloxacin in cohorts A and B and for therapeutic-dose ciprofloxacin in cohort A. However, the AUC_ELF_/AUC_plasma_ ratio was 3.6-fold lower for microdose ciprofloxacin than for therapeutic-dose ciprofloxacin in cohort A, but comparable to that of therapeutic-dose ciprofloxacin in cohort B.

**Table 2:** Comparison of dose-adjusted PK parameters obtained from quantification of microdose ciprofloxacin with accelerator mass spectrometry in cohort A and cohort B and dose-adjusted PK parameters obtained from quantification of therapeutic-dose ciprofloxacin with liquid chromatography–tandem mass spectrometry (cohort A)

	t_max_ (h)	C_max_ (pg equivalents/mL)	AUC (pg equivalents/mL.h)	AUC_microdose_/AUC_therapeutic dose_
Microdose ciprofloxacin (cohort A)				
Plasma	2.00	4.24	19.32	0.8
Subcutaneous tissue	2.00 (2.00–8.00)	1.88 (1.33–2.67)	8.47 (5.46–13.15)	1.0
ELF	8.00	0.76	3.08	0.2
AUC ratios	AUC_ST_/AUC_plasma_		0.44	
	AUC_ELF_/AUC_plasma_		0.16	
Microdose ciprofloxacin (cohort B)				
Plasma	2.00	5.12	21.69	0.9
Subcutaneous tissue	2.00 (2.00–4.00)	2.51 (1.91–3.29)	9.60 (7.55–12.20)	1.1
ELF	2.00	2.98	9.20	0.7
AUC ratios	AUC_ST_/AUC_plasma_		0.44	
	AUC_ELF_/AUC_plasma_		0.42	
Therapeutic-dose ciprofloxacin (cohort A)				
Plasma	1.00 (0.50–1.00)	10.36 (8.66–12.40)	23.26 (20.26–26.70)	
Subcutaneous tissue	2.00 (2.00–2.00)	2.36 (1.43–3.90)	8.81 (5.25–14.78)	
ELF	2.00	4.20	13.41	
AUC ratios	AUC_ST_/AUC_plasma_		0.38	
	AUC_ELF_/AUC_plasma_		0.58	

C_max_ and AUC values are reported as geometric mean with 95% confidence interval in parentheses and t_max_ is reported as median with range in parentheses. For PK parameters of microdose ciprofloxacin in plasma and microdose and therapeutic-dose ciprofloxacin in ELF, no 95% confidence interval is given as only one timepoint per subject was measured and concentrations from three subjects per timepoint were averaged. To enable comparison of microdose and therapeutic-dose ciprofloxacin concentrations, values were normalized to the administered dose and expressed as pg equivalents per mL

*AUC* area under the concentration–time curve (calculated from 0 to 8 h for plasma, subcutaneous tissue and ELF), *AUC*_*ELF*_*/AUC*_*plasma*_ ratio of AUC in ELF to AUC in plasma, *AUC*_*microdose*_*/AUC*_*therapeutic dose*_ ratio of dose-adjusted AUC of microdose ciprofloxacin to dose-adjusted AUC of therapeutic-dose ciprofloxacin, *AUC*_*ST*_*/AUC*_*plasma*_ ratio of AUC in subcutaneous tissue to AUC in plasma, *C*_*max*_ maximum concentration, *ELF* epithelial lining fluid, *PK* pharmacokinetic, *t*_*max*_ time to maximum concentration

## Discussion

This study investigated the feasibility of combining microdosing with two methods for tissue PK determination, that is, microdialysis and BAL. As a model drug, the fluoroquinolone antibiotic ciprofloxacin was chosen. Ciprofloxacin is a biopharmaceutics classification system class IV drug with low permeability, which is a substrate of multiple membrane transporters, which may influence its absorption, tissue distribution, and excretion [34, 35]. Renal clearance accounts for about 70% of the total plasma clearance of ciprofloxacin; the drug undergoes little metabolism and is mainly excreted in an unmetabolized form into urine [36]. Ciprofloxacin has a large volume of distribution (2–3 L/kg) [36] and penetrates well into different tissues. In previous studies, microdialysis [27–29], BAL [37–40], and PET [23, 27] have been used to assess the tissue distribution of ciprofloxacin. In the present study, ciprofloxacin was used in a ^14^C-labeled form to enable AMS quantification and the pharmacokinetics of the [^14^C]ciprofloxacin microdose in plasma, subcutaneous tissue, and ELF was directly compared with that of a standard therapeutic dose of ciprofloxacin quantified with LC-MS/MS. Because of the known metabolic stability of ciprofloxacin and the expected absence of circulating metabolites (i.e., at 2 hours after an i.v. bolus injection of [^18^F]ciprofloxacin, no radiolabeled metabolites could be detected in plasma [23]; after an i.v. infusion of [^18^F]ciprofloxacin, > 85% of total radioactivity excreted into urine over 5 hours was in the form of unchanged [^18^F]ciprofloxacin [27]), total ^14^C was quantified with AMS without prior chromatographic separation and assumed to equal the concentration of [^14^C]ciprofloxacin.

Plasma and subcutaneous PK data of ciprofloxacin after administration of a single i.v. dose of 400 mg (Table 1) were in accordance with previously published data [28, 41]. We found a relatively good agreement between the dose-adjusted plasma and subcutaneous pharmacokinetics of microdose and therapeutic-dose ciprofloxacin (Table 2). Dose-adjusted AUC values of microdose ciprofloxacin in plasma and subcutaneous tissue were within a 0.8-fold to 1.1-fold range of the respective dose-adjusted AUC values of therapeutic-dose ciprofloxacin. However, plasma concentrations based on a total ^14^C analysis with AMS were higher than those based on an LC–MS/MS analysis, in particular at later timepoints (> 4 h) of the experiment, which pointed to a possible contribution of ^14^C-labeled metabolites to total ^14^C content in plasma (Fig. 2a). Interestingly, there was a considerably better agreement between AMS-based and LC–MS/MS-based ciprofloxacin concentrations in subcutaneous tissue (Fig. 2b), which may suggest that radiolabeled metabolites of [^14^C]ciprofloxacin did not penetrate into tissue. Moreover, the pharmacokinetics of [^14^C]ciprofloxacin administered as a microdose only (cohort B) predicted the pharmacokinetics of therapeutic-dose ciprofloxacin equally well as the ^14^C-microdose preceded by the therapeutic dose (cohort A) (Table 2). This is remarkable as it indicates dose linearity of ciprofloxacin pharmacokinetics over the studied large dose range (approximately 360,000-fold). Penetration of microdose ciprofloxacin into subcutaneous tissue was comparable to that of therapeutic-dose ciprofloxacin and AUC_ST_/AUC_plasma_ ratios of microdose ciprofloxacin (cohort A: 0.44, cohort B: 0.44) were in good agreement with the AUC_ST_/AUC_plasma_ ratio of therapeutic-dose ciprofloxacin (0.38) (Table 2).

Epithelial lining fluid concentrations of both therapeutic-dose and microdose ciprofloxacin could be detected up to 4 h after administration, but showed a high inter-subject variability (Fig. 2c). Epithelial lining fluid pharmacokinetics of ciprofloxacin has been assessed before after single and multiple oral doses [37–40], but no data on ELF pharmacokinetics of ciprofloxacin after single i.v. administration have been published until now. As opposed to subcutaneous tissue, penetration of therapeutic-dose ciprofloxacin into ELF was not well predicted by microdose ciprofloxacin with an underprediction of the AUC_ELF_/AUC_plasma_ ratio by a factor of 3.6 in cohort A (Table 2). This suggested that microdosing did not adequately work in combination with BAL, at least for ciprofloxacin. Whether this finding can be ascribed to the applied correction for ELF dilution (urea dilution method) or to reaching limits of detection of [^14^C]ciprofloxacin after dilution of the samples for AMS analysis by addition of ^12^C currently remains unclear. It should be noted that another study successfully used microdosing of an antibiotic drug candidate in combination with BAL to demonstrate drug penetration into different lung compartments in humans [42].

Although microdosing did not provide satisfactory results for determining intrapulmonary pharmacokinetics, this study supports microdosing in combination with microdialysis to determine subcutaneous tissue pharmacokinetics, at least for the investigated model drug ciprofloxacin. This finding could be beneficial for future phase 0 studies, as the possibility to obtain tissue PK data using a minimally invasive method would substantially increase the value of microdose studies. The increasing use of microdialysis for target-site PK determination in early phase I studies or for drug monitoring in clinical practice would provide a perfect situation for combining microdialysis and microdosing. Accordingly, the selection of early drug candidates in phase 0 studies could be accelerated and investigation of tissue penetration in patients could be performed in parallel to standard phase I development.

One limitation of this study is that only total ^14^C content was quantified in plasma samples, which made it impossible to correct the AMS-based concentration measurements for a possible contribution of radiolabeled metabolites of [^14^C]ciprofloxacin. This may be overcome in future studies by performing chromatographic separation of plasma samples prior to AMS analysis. Another limitation is the small sample size and the relatively limited number of samples. Because of ethical reasons but also because it is known that repeated BAL procedures can impact PK assessment in ELF, the procedure was performed only once in each study participant. Therefore, calculation of PK data in ELF was only possible using concentrations of different individuals at several timepoints. A higher sample size would have minimized the inter-individual variability. The fact that pharmacokinetics was assessed only in healthy volunteers and only after a single-dose administration might also limit the generalizability of data but is not expected to have impacted the primary outcome of the study.

## Conclusions

Using the biopharmaceutics classification system class IV drug ciprofloxacin as a model drug, the present data showed that combining microdosing and microdialysis is a feasible concept. The pharmacokinetics of the microdose of [^14^C]ciprofloxacin in plasma and subcutaneous tissue was shown to be predictive of the pharmacokinetics of therapeutic-dose ciprofloxacin. ^14^C-labeled drug product is usually available early during development and integrating microdialysis in microdose studies would add major value to phase 0 studies by providing much needed information on target tissue pharmacokinetics without requiring a local radiolabeling infrastructure. Consequently, time during drug development as well as costs could be reduced, and fewer healthy subjects would be needed to be exposed during phase 1 studies. Phase 0 studies may be an attractive approach during antimicrobial drug development, given the urgent need of new antimicrobial agents in times of increasing antimicrobial resistance. Further studies assessing microdose pharmacokinetics in BAL fluid are warranted to see if the limitations observed in the present study persist.

## Supplementary Information

Below is the link to the electronic supplementary material.

Supplementary file1 (DOCX 40 kb)

## Declarations

### Availability of data and material

Data are available from the corresponding author on reasonable request.
