# Supplementary material for: Microdosing as a Potential Tool to Enhance Clinical Development of Novel Antibiotics: A Tissue and Plasma PK Feasibility Study with Ciprofloxacin
Source: Clin Pharmacokinet. 2022 Jan 7;61(5):697–707. doi: 10.1007/s40262-021-01091-1 (PMC9095552; doi:10.1007/s40262-021-01091-1)
Supplement: Supplementary file 1 — Supplementary file1 (DOCX 40 kb) [file 40262_2021_1091_MOESM1_ESM.docx]

**Electronic Supplementary Material**

Journal: *Clinical Pharmacokinetics*

**Microdosing as a Potential Tool to Enhance Clinical Development of Novel Antibiotics: a Tissue and Plasma PK Feasibility Study**

Zoe Oesterreicher^1,2^, Sabine Eberl^1^, Beatrix Wulkersdorfer^1^, Peter Matzneller^1^, Claudia Eder^1^, Esther van Duijn^3^, Wouter H. J. Vaes^3^, Birgit Reiter^4^, Thomas Stimpfl^4^, Walter Jäger^5^, Alina Nussbaumer-Proell^1^, Daniela Marhofer^6^, Peter Marhofer^6,7^, Oliver Langer^1^, Markus Zeitlinger^1^

^1^ Department of Clinical Pharmacology, Medical University of Vienna, Vienna, Austria

^2^ Internal Medicine 2, Gastroenterology and Hepatology and Rheumatology, University Hospital of St. Pölten, Austria.

^3^ TNO, Zeist, The Netherlands

^4^ Department of Laboratory Medicine, Medical University of Vienna, Vienna, Austria

^5^ Department of Pharmaceutical Sciences, University of Vienna, Vienna, Austria

^6^ Department of Anaesthesia, General Intensive Care and Pain Therapy, Medical University of Vienna, Vienna, Austria

^7^ Orthopaedic Hospital Speising, Vienna, Austria

Corresponding author: Markus Zeitlinger, Department of Clinical Pharmacology, Medical University of Vienna, Währinger Gürtel 18-20, 1090 Vienna, Austria; E-mail: [markus.zeitlinger@meduniwien.ac.at](mailto:markus.zeitlinger@meduniwien.ac.at); Telephone: +43 1 40400 29810, Fax: +43 1 40400 29980.

ORCID: 0000-0002-1873-3953

**Liquid Chromatography Tandem Mass Spectrometry (LC-MS/MS) Analysis**

Ciprofloxacin concentrations in plasma, microdialysis (MD) and bronchoalveolar lavage (BAL) fluid samples were analyzed using LC-MS/MS with a 5500 QTrap system (Sciex, Framingham, MA, USA) equipped with a TurboIon Source for electrospray ionization. As chromatographic system either a Symbiosis ALIAS chromatographic system (Spark Holland B.V., Emmen, The Netherlands) or a Thermo Scientific Ultimate 3000 (Waltham, MA, USA) was used. Ciprofloxacin (Sigma-Aldrich), norfloxacin (Sigma-Aldrich) and d8-ciprofloxacin (Toronto Research Chemicals, Toronto, Canada) were used as standards.

For the analysis of plasma samples, a Kinetex F5 (2.6 µm, 100 Å, 100 x 2.1 mm, Phenomenex, Torrance, CA, USA) was used. Mobile phase A consisted of 0.1% aqueous formic acid (*v*/*v*), mobile phase B of acetonitrile at a flow rate of 0.40 mL/min and a gradient elution program as follows: 90% A with a linear increase up to 90% B over 2.5 min and held for 0.5 min. The mass spectrometer was operated in the positive electrospray ionization mode. Quantification was performed by multiple reaction monitoring. The used mass transitions were for ciprofloxacin m/z 332.0 → 230.9 and for norfloxacin (internal standard) m/z 320.0 → 231.0. Pooled plasma was used for establishing calibrators and quality controls in the concentration range of 0.1 to 10 µg/mL using norfloxacin as internal standard. For sample preparation, 70 µL of plasma and 10 µL of internal standard solution (5 µg/mL norfloxacin in water) were transferred to Eppendorf cups, precipitated with 70 µL of acetonitrile, vortexed for approximately 10 seconds and centrifuged for 5 min at 20,800 *g*. 50 µL of the supernatant was transferred into an autosampler vial, diluted with 150 µL water, vortexed and 2 µL injected into the LC-system. The method for the quantification of ciprofloxacin in plasma was validated according to the EMA Guideline on Bioanalytical Method Validation (EMEA/CHMP/EWP/192217/2009 Rev. 1 Corr. 2**).

Selectivity (blank sample compared to lower limit of quantification, LLOQ) was within the acceptance criteria of 20%. Carry over (blank run after highest calibrator) was above the acceptance criteria of 20% (46%), but was not relevant because of injection of blanks in between samples. LLOQ was determined as the lowest concentration of the calibration curve; results for precision and accuracy can be found in Table S1. The calibration curve was calculated with 1/x weighting and linear regression. All four calibration curves analyzed during validation showed a correlation coefficient of *r* = 0.999. Within-day and between-day precision and accuracy for four quality control (QC) levels (LLOQ - QC, low QC, medium QC and high QC) are shown in Table S1.

**Table S1** Within- and between-day precisions (expressed as coefficient of variation, COV) and accuracies (expressed as bias)

|  | **Within-day precision and accuracy (*n* = 5)** | | | **Between-day precision and accuracy (*n* = 9)** | | |
| --- | --- | --- | --- | --- | --- | --- |
| Nominal concentration (µg/mL) | Mean concentration (µg/mL) | COV (%) | Bias (%) | Mean concentration (µg/mL) | COV (%) | Bias (%) |
| 0.100 | 0.110 | 2.8 | 10.0 | 0.120 | 8.4 | 20.0 |
| 0.300 | 0.306 | 3.6 | 0.0 | 0.300 | 1.3 | 0.0 |
| 4.000 | 4.020 | 2.8 | 0.5 | 4.160 | 5.0 | 4.0 |
| 8.000 | 8.070 | 2.7 | 0.9 | 8.190 | 4.5 | 2.4 |

The matrix effect was tested with six different plasmas spiked with concentrations of low QC and high QC; the coefficient of variation (COV) of matrix effect normalized to internal standard was 13% for low QC and 2% for high QC (acceptance criteria 15%). Short-term temperature stability (2 hours at room temperature) and freeze/thaw stability (3 times thawed to room temperature) was tested with low and high QC and was within the acceptance criteria of 15% (Bias low QC 0.0-6.7%; Bias high QC -3.1-0.75%).

For the determination of ciprofloxacin in MD and BAL fluid samples, the analytical method and sample preparation were modified in order to allow for lower concentrations and samples volumes. For both sample types, a Luna Omega C18 (1.6 µm, 100 Å, 50 x 2.1 mm, Phenomenex, Torrance, CA, USA) was used and d8-ciprofloxacin (internal standard) was analyzed with a mass transition of m/z 340.3 → 296.2.

For MD samples, mobile phase A consisted of 0.1% aqueous formic acid (*v*/*v*) and mobile phase B of 0.1% aqueous formic acid in acetonitrile (*v*/*v*) at a flow rate of 0.55 mL/min and a gradient elution program as follows: 95% A (held for 0.25 min) with a linear increase up to 95% B over 1 min and held for 1.25 min and back to 95% A (held for 2 min). Saline solution was used for establishing calibrators and quality controls. MD samples were analyzed within a concentration range of 50-5500 ng/mL. Samples were prepared as follows: 10 µL of sample and 5 µL of internal standard solution (100 µg/mL in water) were transferred into LC-vials with inserts, diluted with 40 µL of methanol and 200 µL of water, vortexed for approximately 10 seconds and 1 µL was injected into the LC system.

For BAL fluid samples, mobile phase A consisted of 1% aqueous formic acid (*v*/*v*), mobile phase B of acetonitrile at a flow rate of 0.6 mL/min and a gradient elution program as follows: 90% A (held for 0.25 min) with a linear increase up to 90% B over 1 min and held for 1.25 min and back to 90% A (held for 2 min). Saline solution was used for establishing calibrators and quality controls. BAL fluid samples were analyzed within a concentration range of 5-25 ng/mL. Samples were prepared as follows: 50 µL of sample and 10 µL of internal standard solution (100 µg/mL in water) were transferred to Eppendorf cups, precipitated with 25 µL of acetonitrile, vortexed for approximately 10 seconds and centrifuged for 5 min. 50 µL of supernatant were transferred into LC vials with insert, diluted with 25 µL of water and 1 µL was injected into the LC system.
